# Carbon fibre PEEK versus titanium cephalomedullary nails for management of oncological lesions of the femur: a retrospective cohort study

**DOI:** 10.1186/s12891-025-09411-3

**Published:** 2025-12-18

**Authors:** Christian Marx, Tim Cheok, Allan Villadsen, Luis Munoz, Ruurd L Jaarsma, Jakub Jagiello, Luke Johnson

**Affiliations:** 1 Department of Orthopaedic Surgery, Flinders Medical Centre, Bedford Park, South Australia Australia; 2 College of Medicine and Public Health, Flinders University, Bedford Park, South Australia Australia; 3 Department of Orthopaedic Surgery, The Prince Charles Hospital, Chermside, Queensland, Australia; 4 South Australian Sarcoma and Bone Tumour Unit, Flinders Medical Centre, Bedford Park, South Australia Australia; 5 Musculoskeletal Tumour Unit, Rigshospitalet, Copenhagen, Denmark; 6 Genesis Care, Flinders Private Hospital, Bedford Park, South Australia Australia; 7 Department of Orthopaedic Surgery, The Royal Adelaide Hospital, Adelaide, South Australia Australia

**Keywords:** Carbon fibre, Titanium, Intramedullary device, Femur

## Abstract

**Background:**

Carbon fibre reinforced polyetheretherketone (CF-PEEK) intramedullary devices have been gaining traction in the field of orthopaedic oncology. We aim to investigate the safety and efficacy of this device.

**Methods:**

A retrospective cohort study was performed. Our primary objectives were to compare all-cause revision and the incidence of postoperative complications between CF-PEEK nails (Group 1) to titanium alloy nails (Group 2) for management of oncological lesions of the femur. Secondary objectives were to compare estimated blood loss and all-cause mortality between the two groups. Each outcome was adjusted for age, sex, American Society of Anaesthesiologist score and surgical indication (pathological fracture versus prophylactic fixation).

**Results:**

Eighty-two patients with 85 hips (Group 1 = 50; Group 2 = 35) were identified. Our competing risk analysis found no significant difference in the revision risk between groups (subdistribution hazard ratio = 1.14, *p* = 0.931). There was an 85% increase in odds of complication in Group 2 which was not statistically significant (*p* = 0.249). Furthermore, those in Group 2 experienced 306.35 mL less blood loss than Group 1, which was also not statistically significant (*p* = 0.086). There was no difference in all-cause mortality between groups (HR = 1.11, *p* = 0.702).

**Conclusion:**

Our study demonstrates no difference in the safety and efficacy profile of CF-PEEK nails as compared to titanium alloy nails in the management of oncological lesions of the femur. This needs to be interpreted with caution given the underpowered nature of this study. Furthermore, the current implant cost of CF-PEEK nails is prohibitive. Adequately powered randomised controlled trials or multinational registry-based studies incorporating economic assessment and patient reported outcome measures are required to further investigate this new technology prior to its adoption.

**Level of evidence:**

Therapeutic III.

**Supplementary Information:**

The online version contains supplementary material available at 10.1186/s12891-025-09411-3.

## Introduction

Carbon fibre reinforced polymers have a high strength-to-weight ratio and durability, making it an attractive material in the medical device industry [1]. One such example is carbon fibre reinforced polyetheretherketone (CF-PEEK), which comprises of parallel carbon fibre sheets embedded within a polymer matrix of polyetheretherketone [2]. The carbon fibres bear the load, while the polyetheretherketone matrix holds the fibres in place [3]. When used as a material for intramedullary nails, these devices are thought to have improved fatigue properties, which may theoretically reduce the risk of nail breakage, which is a major concern in the treatment of oncological lesions in the long bones [4].

Several previous studies have demonstrated that the median survivorship in patients with metastatic bone disease was less than a year [5–7], with those treated for an impending fracture rather than completed fracture having a better prognosis [8–10]. The treatment of femoral metastases is therefore of palliative intent, with a goal to provide pain relief, improve quality of life, and facilitate early mobilisation [11, 12]. Depending on patient and injury characteristics, this can be achieved with either intramedullary devices or endoprosthesis reconstruction [13–17]. A recent audit in the United Kingdom have found that intramedullary fixation was the most popular option for management of this condition [18]. The emergence of radiolucent CF-PEEK nails provide an attractive alternative to the traditional titanium alloy nails for stabilisation of these lesions, where cephalomedullary nails are indicated [19]. Early introductory studies have found acceptable clinical outcomes of these devices, sparking further research in this area [20, 21].

Despite the femur being the most common site for long bone metastasis [22], there are no dedicated study comparing the safety and efficacy of CF-PEEK nails to titanium alloy nails for stabilisation of this. In addition to their superior fatigue properties, these radiolucent devices allow for continued surveillance of oncological lesions and improves the delivery of radiotherapy. Of interest, several previous studies have indicated no significant differences between the two implants. However, all these studies included a variety of long bones, thus casting doubts over the validity of the conclusions drawn [4, 23–26]. In this study, our primary aim is to compare all-cause revision and the incidence of postoperative complications between CF-PEEK nails to titanium alloy nails for management of oncological lesions of the femur. Secondary outcomes of interest were to compare estimated blood loss and all-cause mortality between the two groups.

## Materials and methods

This is a retrospective cohort study reported in accordance with the Strengthening the Reporting of Observational Studies in Epidemiology (STROBE) guidelines [27]. Human research ethics committee approval was sought and obtained from the Southern Adelaide Clinical Human Research Ethics Committee prior to commencement of this study (Approval No.: EC00188/20.25).

### Patient selection

A systematic search of the South Australian Sarcoma and Bone Tumour Unit database was performed to identify consecutive adult patients (above 18 years of age) with cephalomedullary nails management of oncological lesions of the femoral diaphysis (shaft) between 1st of January 2016 and 31st of January 2024 was performed. Our unit was established in 2015 as an integrated multidisciplinary system in South Australia and acts as the central point of referral for all patients with suspected or confirmed bone and soft tissue tumours within our state. We excluded patients who had stainless steel devices, those below the age of 18, and those who were treated for non-femoral lesions.

The patients were divided into two groups; Group 1 comprised of patients who were managed with long CF-PEEK nails, and Group 2 comprised of patients who were managed with long titanium alloy nails. Within the framework of our service, all patients in Group 1 received a CarboFix Trochanteric (PF) Nail (CarboFix Orthopaedics Ltd, Herzeliya, Israel), whereas patients in Group 2 received either a Long Gamma Nail (Stryker GmbH, Selzach, Switzerland), TRIGEN META-TAN Trochanteric Antegrade Nail (Smith & Nephew Inc, Tennessee, USA) or AFFIXUS Hip Fracture Nail (Biomet Trauma, Indiana, USA). The choice of implant was left to the discretion of the treating surgeon. Procedures were carried out in the local hospital deemed most suitable for the individual patient, which was usually incumbent upon staff and equipment availability. No cement augmentation was used in these cases, and the nail was placed in static mode with two distal screws. The primary surgeons for these procedures were experienced with the technique for conventional cephalomedullary nail insertion. All patients were followed up until death, or in the case of survival, until the 8th of November 2024.

### Data collection, outcomes and statistical analysis

Relevant baseline information was extracted from medical records including age at diagnosis, sex, body mass index (BMI), primary tumour, presence of pathological fracture, American Society of Anaesthesiologists (ASA) score, extent of disease, concurrent chemotherapy or immunotherapy, preoperative radiotherapy to the femur, as well as baseline haematological and biochemical markers. The primary outcomes of interest were all-cause revision and the incidence of postoperative complications. Revision was defined as any secondary procedure involving removal and/ or exchange of the cephalomedullary nail at any time frame between the patients’ index procedure until death. Postoperative complication was defined as Clavien-Dindo grade 3 within 30 days of their index procedure and/ or presence of acute kidney injury within two weeks of surgery. In the analysis of all-cause revision, Fine and Gray competing risk survival analysis was performed due to the high mortality rate, with the groups compared using subdistribution hazard ratios (SHR). The incidence of postoperative complications was compared using odds ratios (OR).

Secondary outcomes of interest were estimated blood loss and all-cause mortality. Blood volume was first calculated using the technique described by Nadler et al., followed by estimation of blood loss using the formula described by Bourke et al. [28, 29]. Difference in estimated blood loss was compared using linear regression. Mortality was compared between groups using Kaplan-Meier survival functions, as well as hazard ratios (HR) derived from Cox-regression analysis. Each outcome was adjusted for age, sex, ASA score and surgical indication (prophylactic fixation versus pathological fracture) using complete case analysis. For continuous data, we utilised a linear regression model, whereas in discrete data, a penalised logistic regression model as described by Firth was utilised [30]. Continuous variables were displayed as median (range) and compared using the Mann-Whitney U test, whereas discrete variables were displayed as count (percentage) and compared using Chi-squared test. Those with bilateral disease were analysed separately. Sensitivity analysis was performed for each hospital site to ensure that no significant hospital site accounted for variation in outcomes. Similarly, we analysed the same outcomes based on the type of titanium nails to ensure that the outcomes were not biased due to within-device heterogeneity. All statistical analysis was performed using Stata Version 18.0 (StataCorp LLC, Texas, USA). Threshold for statistical significance was set at *p* < 0.050.

## Results

Using our inclusion and exclusion criteria, we identified eighty-five cephalomedullary nails that were inserted into 82 patients, with three cases of bilateral disease. Flowchart for selection is displayed in Supplementary Fig. 1. There were forty-eight patients (50 femurs) in Group 1 and thirty-four patients (35 femurs) in Group 2. Of the 35 femurs included in Group 2, 60.0% were Long Gamma Nails, 28.6% were TRIGEN META-TAN Trochanteric Antegrade Nails, and the remaining 11.4% received an AFFIXUS Hip Fracture Nail. The median age at time of surgery was 70.21 years for Group 1 and 68.70 years for Group 2, with majority of patients being female in both groups. These were not statistically significant. There was also no statistically significant difference in BMI (*p* = 0.453) and distribution of ASA scores (*p* = 0.520) between groups. Average follow-up time was 1.1 years, with no loss to follow-up.

The most common primary tumour in Group 1 was breast cancer (24.0%), whereas in Group 2 it was lung cancer (28.6%). This difference in primary tumour distribution was statistically significant (*p* = 0.003). Nevertheless, there were no significant difference in terms of surgical indication (*p* = 0.082), with prophylactic fixation being more common than pathological fracture in both groups. We found no significant difference in terms of metastatic burden to the viscera (*p* = 0.278), brain (*p* = 0.448), other bones (*p* = 0.100), and spine (*p* = 0.519). Almost all patients in Group 2 had lymph node metastasis, which was significantly higher than Group 1 (*p* = 0.001). There was a significantly higher proportion of patients receiving chemotherapy (*p* = 0.019) and/ or immunotherapy (*p* = 0.001) in Group 1 preoperatively, however no difference in proportion of patients receiving preoperative radiotherapy to the femur. In both groups, most patients had hypalbuminaemia, with a median level of 2.90 g/dL for Group 1 and 2.80 g/dL for Group 2. The median alkaline phosphatase levels were increased for both groups, but lactate dehydrogenase levels were within normal range. There were no statistically significant differences in the baseline haematological and biochemical characteristics between groups. A summary of patient characteristics is shown in Table 1.

**Table 1:** Patient demographic

										Group 1 (*n* = 50)	Group 2 (*n* = 35)	*p*-Value
Age at Index Surgery, Years										70.21 (35.54–84.81)	68.70 (43.58–87.35)	0.591
Sex												0.332
	Male									19 (38.0)	17 (48.6)	
	Female									31 (62.0)	18 (51.4)	
American Society of Anaesthesiologists Score												0.520
	2									2 (4.0)	0 (0)	
	3									24 (48.0)	19 (54.3)	
	4									23 (46.0)	16 (45.7)	
	5									1 (2.0)	0 (0)	
Body Mass Index, kg/m^2^										26.31 (16.65–40.75)	24.34 (17.94–41.41)	0.453
Primary Tumour												**0.003**
					Lung					7 (14.0)	10 (28.6)	
									Small Cell Lung Cancer	6	4	
									Non-Small Cell Lung Cancer	1	6	
					Breast					12 (24.0)	5 (14.3)	
					Prostate					5 (10.0)	3 (8.6)	
					Gastrointestinal					8 (16.0)	5 (14.3)	
								Oropharyngeal		2	0	
								Oesophageal/ Gastric/ Small Bowel		2	0	
								Colorectal		3	3	
								Anal		0	1	
								Cholangiocarcinoma		1	1	
					Haematological					9 (18.0)	8 (22.9)	
							Lymphoma			1	4	
							Multiple Myeloma			8	4	
					Melanoma					3 (6.0)	0 (0)	
					Unknown Primary					1 (2.0)	3 (8.6)	
					Other					5 (10.0)	1 (2.9)	
Indication												0.082
		Prophylactic Fixation								35 (70.0)	18 (51.4)	
		Pathological Fracture								15 (30.0)	17 (48.6)	
Metastatic Burden												
				Presence of Visceral Metastasis						30 (60.0)	25 (71.4)	0.278
				Presence of Lymph Node Metastasis						29 (58.0)	32 (91.4)	**0.001**
				Presence of Brain Metastasis						38 (76.0)	24 (68.6)	0.448
				Presence of Other Bony Metastasis						11 (22.0)	3 (8.6)	0.100
				Spinal Metastasis								0.519
						None				16 (32.0)	15 (42.9)	
						Single Level				8 (16.0)	6 (17.1)	
						Multi-Level				26 (52.0)	14 (40.0)	
Treatment												
			Chemotherapy							38 (76.0)	18 (51.4)	**0.019**
			Immunotherapy							19 (38.0)	2 (5.7)	**0.001**
			Preoperative Radiotherapy to Affected Femur							11 (22.0)	5 (14.3)	0.371
Preoperative Blood Parameters												
	Haemoglobin, g/dL									10.85 (7.70–15.70)	10.9 (7.80–14.90)	0.689
	Albumin, g/dL									2.90 (1.50–4.00)	2.80 (1.90–4.00)	0.185
	Alkaline Phosphatase, IU									109.50 (33.00–1,018.00)	108.00 (18.00–1,426.00)	0.920
	Sodium, mmol/L									138.00 (129.00–143.00)	136.00 (128.00–144.00)	0.361
	Lactate Dehydrogenase, U/L									258.00 (142.00–2,071.00)	265.00 (166.00–2,261.00)	0.495

### Primary outcomes

Of the eighty-five cephalomedullary nails, two patients underwent a revision, one from each group. The patient that was revised in Group 1 underwent a nail exchange for implant breakage a year after her index surgery. Similarly, the second patient from Group 2 underwent a proximal femur replacement due to fracture non-union with some evidence of disease progression two years after the initial surgery. Our adjusted analysis suggested an increased all-cause revision risk in Group 2 over Group 1, which was not statistically significant, with death accounted for as a competing risk (SHR = 1.14, 95% Confidence Interval (CI): 0.06–23.28, *p* = 0.931). The assumption of proportionality was confirmed (*p* = 0.222).

There were two further surgical complications of note, both occurring in Group 2. One patient was found to have a delayed union with impending implant failure approximately 3 months after fixation. However, at that review, it was also noted that the patient had experienced overall disease progression and was palliated. Surgical intervention was thus not offered, and she passed away a week after that review. The second patient had a superficial wound infection and received oral antibiotics. On further review, this had settled down and there were no concerns of deep infection.

Furthermore, we also experienced a few medical complications. One patient in Group 1 had an intraoperative cardiac arrest and passed away. Two patients, one from each group, was found to have a pulmonary embolism, which was managed with anti-coagulation. Postoperative acute kidney injuries occurred in 12.0% of patients in Group 1 and 22.9% of patients in Group 2. Overall, we found no difference in the complication rate between groups, with an adjusted OR of 1.85 (95% CI: 0.65–5.25), *p* = 0.249). A summary of our complications is detailed in Supplementary Table 1.

### Secondary outcomes

The median estimated blood loss was 489.20 mL in Group 1 and 195.20 mL in Group 2. This was not statistically significant (*p* = 0.121). When adjusted for age, gender and ASA score, patients in Group 2 experienced 306.35 mL less blood loss than Group 1. This was also not statistically significant (95%. CI: -657.15–44.45, *p* = 0.086).

**Fig. 1:**
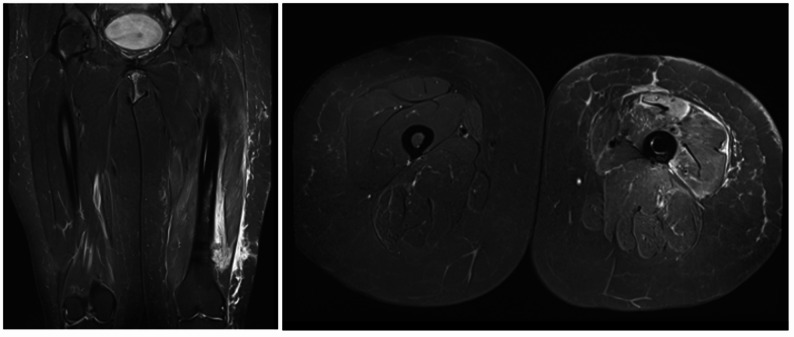
Kaplan-Meier survival analysis for all-cause mortality. Adjusted hazard ratio was 1.11 which was not statistically significant (*p* = 0.702). CF-PEEK = carbon fibre reinforced polyetheretherketone

A substantial proportion of patients had died at time of censorship – 82.0% in Group 1 and 91.4% in Group 2. The median survival time for our entire cohort was 0.38 years. When stratified by group, the median survival time was 0.47 years in Group 1 and 0.26 years in Group 2. The Kaplan-Meier survival function is shown in Fig. 1. Adjusted Cox-regression analysis showed a slightly higher risk of all-cause mortality in Group 2 compared to Group 1 which was not statistically significant (HR = 1.11, 95% CI: 0.65–1.92, *p* = 0.702). The assumption of proportionality was confirmed (*p* = 0.353). The ninety-day survivorship was 64.0% in Group 1 and 57.1% in Group 2, whereas the one-year survivorship was 38.0% in Group 1 and 31.4% in Group 2. These were not statistically different, with an adjusted OR of 0.61 (95% CI: 0.22–1.72, *p* = 0.352) and 0.68 (95% CI: 0.26–1.78, *p* = 0.436) respectively. Our primary and secondary outcomes are shown in Table 2.

**Table 2:** Outcomes Summary. SHR = subdistribution hazard ratio; OR = odds ratio; HR = hazard ratio; β = regression coefficient; CI = confidence interval

	Unadjusted Analysis			Adjusted Analysis		
	Outcome	95% CI	*p*-Value	Outcome	95% CI	*p*-Value
Primary Outcomes						
All-Cause Revision	SHR = 1.31	0.09–19.52	0.845	SHR = 1.14	0.06–23.28	0.931
Complications	OR = 2.35	0.85–6.49	0.100	OR = 1.85	0.65–5.25	0.249
Secondary Outcomes						
Estimated Blood Loss, mL	β = -252.28	-586.70–82.14	0.137	β = -306.35	-657.15–44.45	0.086
All-Cause Mortality	HR = 1.08	0.67–1.76	0.747	HR = 1.11	0.65–1.92	0.702

### Sensitivity analysis

Our sensitivity analysis found no substantial difference in outcomes attributed to the site in which the surgery was performed. If further adjusted for hospital site, the SHR for all-cause revision was 1.07 (95% CI: 0.17–6.82, *p* = 0.942); adjusted OR for complication rates was 3.45 (95% CI: 0.97–12.32, *p* = 0.056); adjusted EBL was − 253.93 mL (95% CI: -746.98–239.11, *p* = 0.306), and; adjusted HR for all-cause mortality was 1.26 (95% CI: − 0.69–23.02, *p* = 0.876). Further analysis on the effect of type of titanium nail on all-cause revision was not possible, due to low revision rates. The only revision in Group 2 received a Long Gamma Nail. The remaining analyses did not reveal any significant difference between the type of titanium nail and the investigated outcomes. Compared to the AFFIXUS Hip Fracture Nail, the adjusted relative risk of complication was 1.59 for the Long Gamma Nail (95% CI: 0.13–19.58, *p* = 0.718) and 1.25 for the TRIGEN META-TAN Trochanteric Antegrade Nail (95% CI: 0.07–23.71, *p* = 0.883); the adjusted estimated blood loss was − 200.71 mL for the Long Gamma Nail (95% CI: -1,803.82–1,402.40, *p* = 0.794) and − 104.10 mL for the TRIGEN META-TAN Trochanteric Antegrade Nail (95% CI: -1,732.33–1,524.14, *p* = 0.894), and; the adjusted HR for all-cause mortality 0.65 for the Long Gamma Nail (95% CI: 0.19–2.16, *p* = 0.479) and 0.29 for the TRIGEN META-TAN Trochanteric Antegrade Nail (95% CI: 0.07–1.21, *p* = 0.088).

## Discussion

This study is a first in evaluating the safety and efficacy of CF-PEEK nails specifically for femoral lesions. In our study, we found no significant difference in all-cause revision, complications, estimated blood loss and all-cause mortality between patients with oncological lesions of the femur treated with CF-PEEK nails versus those managed with titanium alloy nails. When compared to previous studies which had evaluated this in the context of carbon fibre implants in general, our results are consistent with those studies [4, 23–26, 31]. Of interest, despite more patients in the CF-PEEK nail group receiving preoperative chemotherapy and/ or immunotherapy, we did not observe any difference in complication rates. Previous studies have yielded conflicting results on the effect of such treatments on wound healing [32–36]. It is likely that that the higher rates of preoperative chemotherapy/ immunotherapy in Group 1 were due to the differences in the type of primary tumour, rather than a difference in clinical practice. As mentioned previously, the primary purpose of intramedullary nailing for femoral oncologic lesions is to provide pain relief, improvement of quality of life, and facilitate early mobilisation. Takashima et al. have shown that CF-PEEK nails were able to achieve these technical objectives [20]. Although these metrices were not investigated in the present study, a previous studies have found no difference in pain scores, functional outcomes and quality-of-life measures between CF-PEEK and conventional titanium nails [37, 38].

**Fig. 2:**
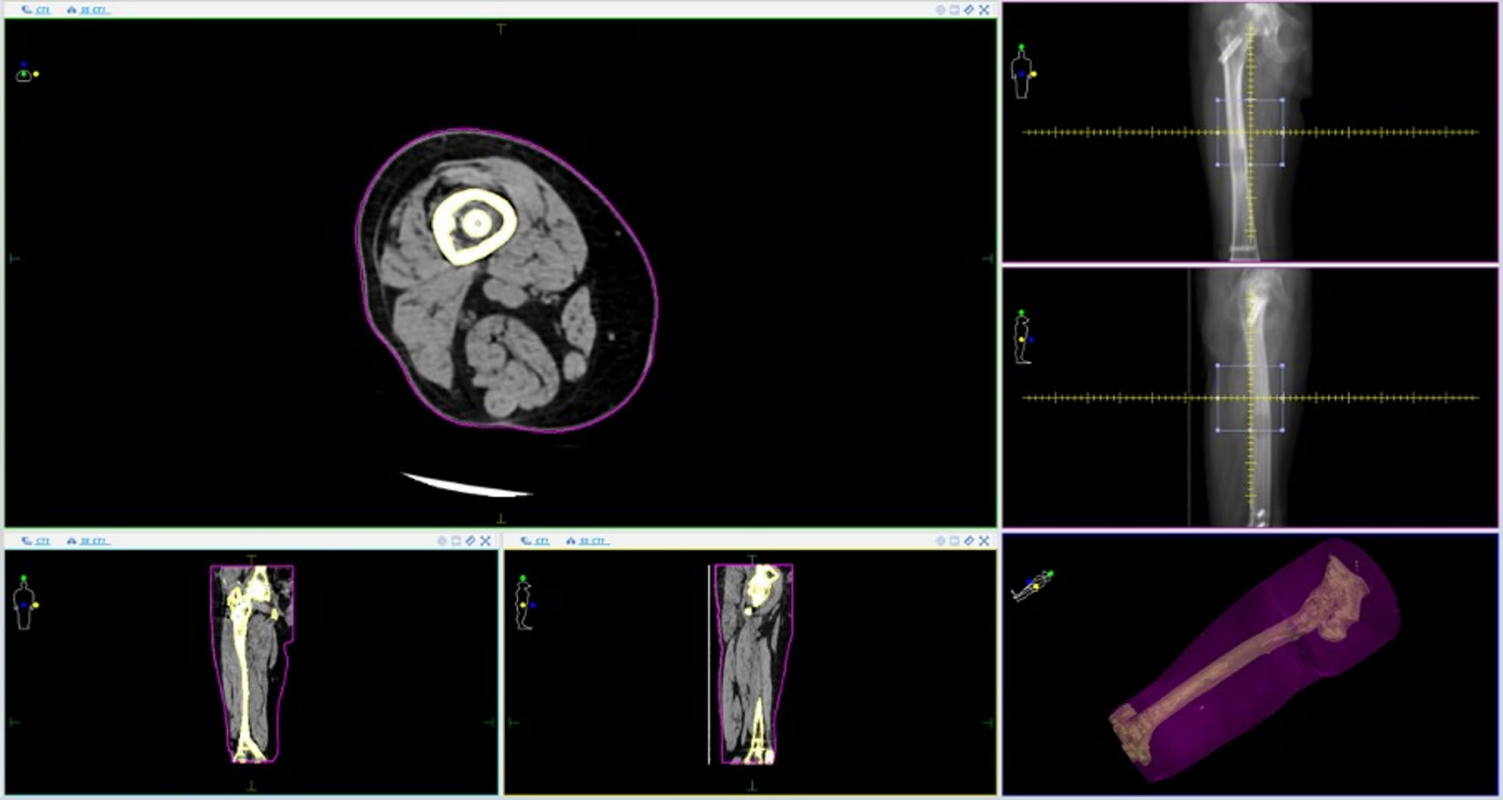
Cross sectional imaging demonstrating radiation therapy planning following carbon fibre reinforced polyetheretherketone (CF-PEEK) nail insertion. The CF-PEEK nail is within the medullary canal of the bone. Figure on top left shows an axial Computed Tomography (CT) of the thigh, corresponding to the area in the top two images on the right. Note minimal scatter in the CT scan despite presence of the cephalomedullary device, allowing for a more targeted approach. The bottom left-most image is a coronal slice, whereas the second image from the bottom left is a sagittal slice, both from the same region described. Region highlighted by the yellow lines indicate bone/ implant, whereas purple line suggests soft tissue

**Fig. 3:**
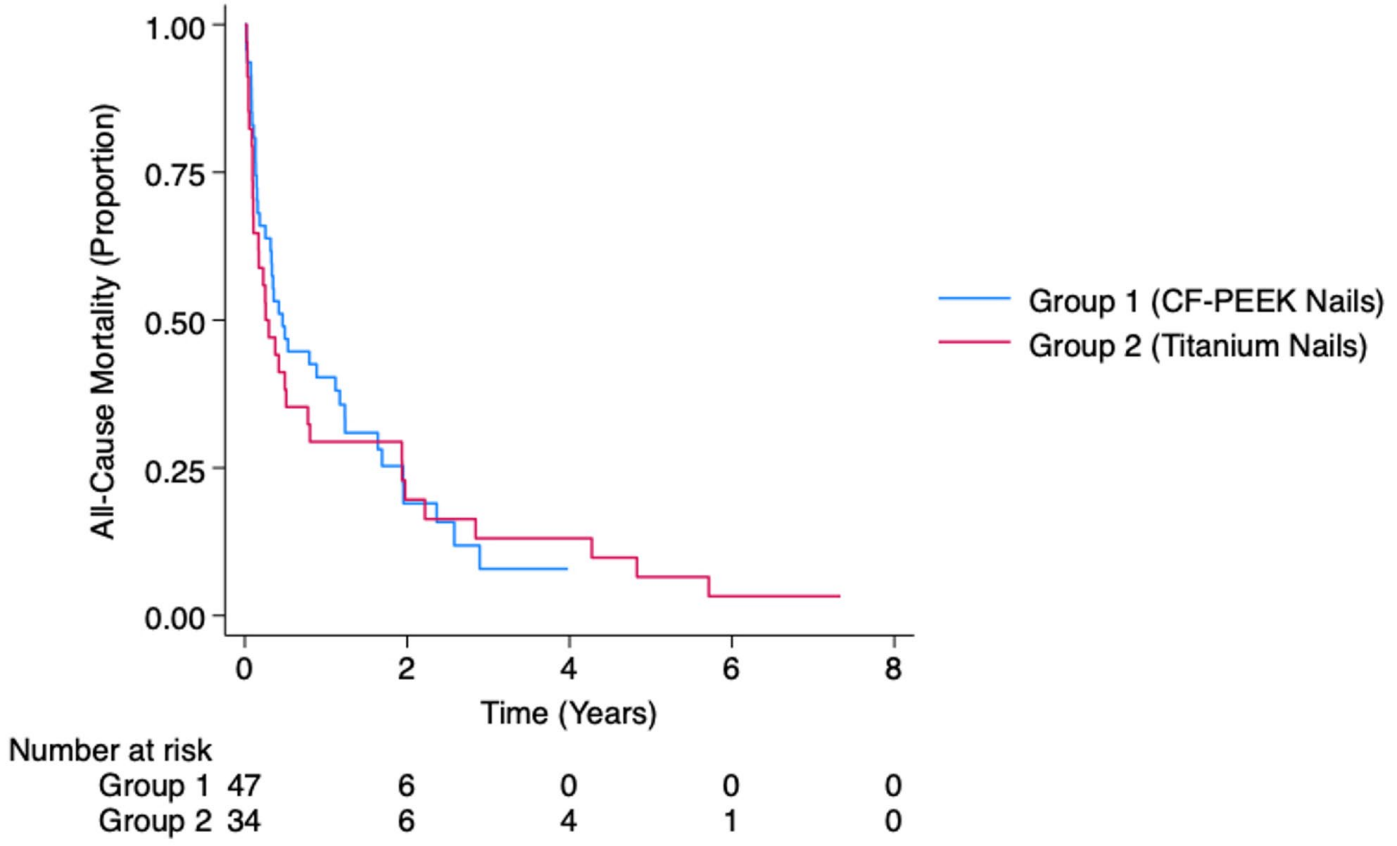
Surveillance Magnetic Resonance Imaging (MRI) for a patient with a history of myxoid liposarcoma who had received a carbon fibre reinforced polyetheretherketone (CF-PEEK) nail for prophylactic fixation following wide local excision of the tumour. The CF-PEEK nail is within the medullary canal of the bone. The image on the left is a coronal slice, whereas the image on the right shows an axial slice of the same region. Note the lack of artifact on both images, despite presence of the cephalomedullary nail

Despite this study demonstrating similar outcomes between CF-PEEK and conventional titanium nails, there are several practical advantages of CF-PEEK nails. Firstly, their radiolucent properties allow for improved quality of follow-up radiographs and computed tomography (CT) imaging [39, 40]. This in turn permits better surveillance for fracture healing and aids radiation oncologists in planning appropriate radiotherapy for these patients (Fig. 2) [41]. Secondly, these implants are also non-magnetic, allowing for high quality surveillance magnetic resonance imaging (MRI), especially in the context of sarcomas (Fig. 3) [42]. Thirdly, the low attenuation and scattering properties of these implants facilitate effective delivery of radiotherapy doses to the lesion while minimising irradiation of adjacent soft tissue structures [43, 44]. The reduced imaging artifacts associated with CF-PEEK implants enable clearer visualization of adjacent lesions, facilitating precise targeting during treatment planning and potentially improving clinical outcomes. This is particularly important in the of management of large volume soft tissue sarcoma of the thigh. The nail reinforces the bone when radiation treatment is employed as part of the overall treatment strategy, to protect against late radiation failure of bone. Additionally, there is emerging evidence that such implants may be used for targeted delivery of chemotherapeutic agents [45]. Another proposed benefit of the CF-PEEK implant is the favourable biomechanical properties of this material [46]. It is possible that the use of CF-PEEK implants in situations where there is poor healing potential, such as pathological femur fractures, reduces the risk of catastrophic implant failure. Biomechanical studies of CF-PEEK nails in this context is being carried out in our institution. In our study, neither group experienced a catastrophic implant failure. We postulate that this may be due to the high mortality rate in our patient cohort, and therefore larger studies are required. Of further interest, a photodynamic intramedullary nailing system has recently been introduced as a potential treatment for pathological lesions [47]. Nevertheless, a recent clinical study has found a high implant fracture rate when used for management of fractures [48], and thus intramedullary devices should be considered in this instance.

We acknowledge several limitations of this study. The retrospective design of this study relies upon accurate recording at time of admission. It is possible that minor complications, such as superficial wound dehiscence was not accurately documented in the patient record. However, major complications (Clavien-Dindo Grade 3 and above) and presence of acute kidney injury was well recorded and thus presented in this study. Secondly, although this is the largest series to date, the study is still underpowered. *Post-hoc* sample size calculation found that for a hazard ratio of 1.14, 919 patients are required in each group to achieve a power of 80%, assuming α = 0.05. Thirdly, despite adjusting for various factors, there was still a difference in the primary tumour distribution, presence of nodal metastases, as well as concurrent chemotherapy or immunotherapy between the two groups. Due to our limited failure rate, we were unable to fully adjust for these factors, thus risking residual confounding. Fourth, the use of an equation-based method for estimation of blood loss may be criticised. Although it is generally accepted that equation-based methods are more objective and accurate than intraoperative estimation methods [49, 50], controversy still exists on the specific equation which should be used [51–53]. In this study, we utilised the Nadler-Bourke equations due to its high precision, as shown in a study by Cumpston [54]. Fifth, as there was no standardised protocol for selection into either group, there is likely to have been a degree of selection bias. Sixth, as with implementation of any new device in orthopaedics, there is bound to be a learning curve effect [55, 56]. In our experience, the technique for insertion of distal locking screws was more technically challenging for CF-PEEK nails due to the radiolucency of the implant. Additionally, there have been case reports highlighting the difficulty in implant retrieval in cases of failure [57]. The median estimated blood loss in the CF-PEEK nail group was higher than in the titanium alloy nail group, although this did not achieve statistical significance. Ideally, operative and fluoroscopic time should be used as a surrogate for procedural complexity. Unfortunately, this is poorly recorded in our service. There were also differences in patient demographics between the two groups, in which we have attempted to address by regression-mediated correction. Furthermore, although in-vivo studies conducted on animals suggested safety of CF-PEEK implants [58, 59], there is a lack of human retrieval studies to confirm these findings. We also did not record preoperative and postoperative pain and functional/ mobility levels, which are equally important surgical goals. This is an important limitation. Lastly, the carbon fibre PEEK nail is substantially more costly, by approximately A$ 1,000, than standard titanium cephalomedullary nail.

In conclusion, our results demonstrate no difference in the safety and efficacy profile of CF-PEEK nails as compared to titanium alloy nails in the management of oncological lesions of the femur. This needs to be interpreted with caution given the underpowered nature of this study. CF-PEEK nails may afford several practical advantages, including improved radiographical surveillance, radiotherapy planning and delivery, although further studies with artifact metrics, dosimetry endpoints and healing visibility scores are required to quantify this. These results are likely to be generalisable. Nevertheless, widespread use is not practical given the substantial increase in cost given the comparable clinical outcomes. Adequately powered randomised controlled trials or multinational registry-based studies, incorporating health economic assessments and patient reported outcome measures, are required to further investigate this new technology prior to its adoption.

## Supplementary Information

Supplementary Material 1: Supplementary Fig. 1.

Supplementary Material 2: Supplementary Table 1.

Supplementary Material 3.

## Data Availability

The datasets used and/or analysed during the current study are available from the corresponding author on reasonable request.
