# Supplementary figures and images for: Carbon fibre PEEK versus titanium cephalomedullary nails for management of oncological lesions of the femur: a retrospective cohort study
Source: BMC Musculoskelet Disord. 2025 Dec 18;27:54. doi: 10.1186/s12891-025-09411-3 (PMC12831453; doi:10.1186/s12891-025-09411-3)

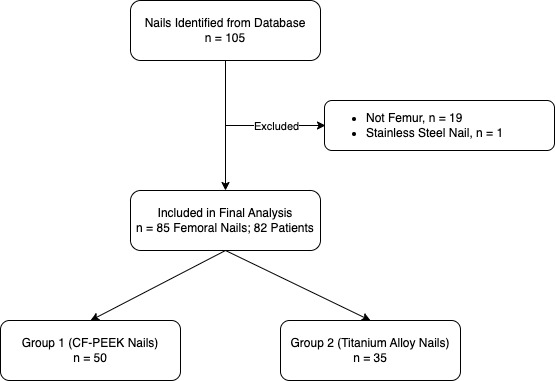

Supplement: Supplementary file 1 — Supplementary Material 1: Supplementary Fig. 1. [file 12891_2025_9411_MOESM1_ESM.jpg]
