# Supplementary material for: Carbon fibre PEEK versus titanium cephalomedullary nails for management of oncological lesions of the femur: a retrospective cohort study
Source: BMC Musculoskelet Disord. 2025 Dec 18;27:54. doi: 10.1186/s12891-025-09411-3 (PMC12831453; doi:10.1186/s12891-025-09411-3)
Supplement: Supplementary file 2 — Supplementary Material 2: Supplementary Table 1. [file 12891_2025_9411_MOESM2_ESM.docx]

Supplementary Table 1: Complications (*patients may have more than one complication)

|  | | Group 1 (n = 50) | Group 2 (n = 35) |
| --- | --- | --- | --- |
| Surgical | |  |  |
|  | Delayed/ Non-union | 1 | 2 |
|  | Superficial Infection | 0 | 1 |
| Medical | |  |  |
|  | Intraoperative Death (Cardiac Arrest) | 1 | 0 |
|  | Pulmonary Embolism | 1 | 1 |
|  | Acute Kidney Injury | 6 | 8 |
| Total Patients with Complications* | | 8 (16.0) | 11 (31.4) |
